# Safety and tolerance assessment of milk fat globule membrane-enriched infant formulas in healthy term Chinese infants: a randomised multicenter controlled trial

**DOI:** 10.1186/s12887-022-03507-8

**Published:** 2022-08-03

**Authors:** BoWen Jiang, Yong Xia, LiHong Zhou, XiaoYing Liang, XuHui Chen, MeiZhen Chen, XiaoXia Li, Shan Lin, Nai Zhang, Ling Zheng, Miao Tao, Peter Petocz, Sophie Gallier, Angela Rowan, Bing Wang

**Affiliations:** 1 Maternal &, Child Health Hospital of Fuzhou, Fuzhou, 350005 China; 2 School of Medicine, Xiamen University, Xiamen City, 361005 China; 3 Maternal &, Child Health Hospital of Fuqing, Fuqing, 350300 China; 4 Changle Hospital, Changle, 350200 China; 5 Second Hospital of Fuzhou, Fuzhou, 350007 China; 6 Macquarie University, Sydney, NSW 2109 Australia; 7 Fonterra Co-Operative Group Limited, Wellington, New Zealand

**Keywords:** Infant feeding, Formula, Neurodevelopment, Milk fat globule membrane, Growth, Formula tolerance

## Abstract

**Background:**

Milk fat globule membrane (MFGM), natural to breast milk, is essential for neonatal development, but lacking from standard infant formulas.

**Objectives:**

To evaluate the safety and tolerability of MFGM supplementation in formula for infants 0 to 12 months.

**Methods:**

In a prospective, multicentre, double-blind, randomized trial, healthy term infants were randomized to a standard formula (SF, *n* = 104) or an MFGM-enriched formula (MF, *n* = 108) for 6 months and a corresponding follow-on formula until 12 months. Exclusively breast-fed infants (*n* = 206) were recruited as the reference group (BFR). Tolerance and safety events were recorded continuously. Anthropometric measurements were assessed at enrolment, 42 days and 4, 6, 8 and 12 months.

**Results:**

Infants (*n* = 375) completed the study with average dropout of < 20%. Stool frequency, color, and consistency between SF and MF were not significantly different throughout, except the incidence of loose stools in MF at 6 months being lower than for SF (odds ratio 0.216, *P* < 0.05) and the frequency of green-colored stools at 12 months being higher in MF (CI 95%, odds ratio 8.92, *P* < 0.05). The BFR had a higher frequency of golden stools and lower rate of green stools (4–6 months) than the two formula-fed groups (*P* < 0.05). SF displayed more diarrhoea (4.8%) than MF (1%) and BFR (1%) at the 8-month visit (*P* < 0.05). BFR (0–1%) had significantly less (*P* < 0.05) lower respiratory infections than MF (4.6–6.5%) and SF (2.9–5.8%) at 6- and 8-months, respectively. Formula intake, frequency of spit-up/vomiting or poor sleep were similar between SF and MF. Growth rate (g/day) was similar at 4, 6, 8 and 12 months between the 3 groups, but growth rate for BFR was significantly higher than for SF and MF at 42 days (95% CI, *P* = 0.001).

**Conclusions:**

MFGM-enriched formula was safe and well-tolerated in healthy term infants between 0 and 12 months, and total incidences of adverse events were similar to that for the SF group. A few differences in formula tolerance were observed, however these differences were not in any way related to poor growth.

## Background

The milk fat globule membrane (MFGM) is a tri-layer of phospholipids, sphingolipids, gangliosides, and cholesterol interspersed with membrane-bound proteins, including glycoproteins, and is secreted by the lactating cells of humans and other mammals [1, 2]. The proteins associated with the MFGM represent up to 1–4% of the total milk proteins [3]. MFGM also contains one or more sialic acid as building blocks of gangliosides [4]. Gangliosides constitute about 6—10% of the total lipid mass of the human brain, where they represent a quarter of the total conjugated saccharides and 70—80% of the conjugated sialic acids [4, 5]. Brain gangliosides play a critical role in neurodevelopment and cognitive function [6, 7]. Ganglioside composition of the intestinal brush border and apical surface of the colon influences numerous cell processes including microbial attachment, cell division, differentiation, and signaling [8]. Dietary MFGM supplementation increases cognitive scores in neonatal animal models and human infants and helps formula-fed infants to reach similar developmental outcomes to breastfed infants [9, 10]. Recent studies have shown that MFGM supplementation promotes development of the intestinal epithelium and microbiome and protects against inflammation [2, 11] through the provision of important components, and/or regulating various cellular events during infant growth and immune education [2, 12, 13]. MFGM supplementation also alleviates inflammation in low-birth-weight mice treated with lipopolysaccharide [13]. However, MFGM supplementation showed no significant effects on weight gain and feed intake of normal birth weight mice during early life and adulthood [2].

Human breast milk is the natural first food for babies and contains all the essential nutrients including a rich supply of MFGM. Milk lipids are secreted in a unique manner by lactocytes, which are specialized epithelial cells within the alveoli of the lactating mammary gland [14]. The lipid fraction of breast milk represents a major energy source for the newborn. The concentration of MFGM phospholipids in human milk is reported to be around 0.5 g/L or 1.5 g/100 g milk fat [15]. In milk, gangliosides are almost exclusively associated with the MFGM [16, 17]. The total amount of gangliosides in human colostrum and mature human milk is reported to be about 26.8 mg/L and 13.1–25.3 mg/L, respective [18, 19]. In contrast, the total amount of gangliosides in mature bovine milk is significantly lower (4.4—6 mg/L) [18, 20]. Gangliosides play an important role in maintaining the integrity of lipid rafts [21] and the modulation of membrane proteins and ion channels, in cell signaling and in communication among cells [6, 22].

Human milk is the gold standard for the promotion of optimal growth and development of infants. However, most infant formulas do not contain MFGM, as the main lipid source is derived from vegetable oils emulsified with milk proteins; this results in a lipid size, structure and composition different to that of human milk fat globules [23]. To ensure the suitability of an infant formula as the sole source of nutrition for infants who cannot be breastfed, an infant starter formula and a follow-on formula have been formulated with ingredients enriched in MFGM to give similar levels of MFGM components to those found in human milk, in order to provide benefits similar to outcomes in breastfed infants.

The present study is part of a randomized controlled trial with the primary aim to evaluate the effects of 12-month supplementation of term infants with bovine MFGM on neurodevelopmental outcome measured at 12 months of agel [24]. In our recent report, the MFGM-enriched infant formula (MF) improved some measures of cognitive development in Chinese infants [24]. The aim of the present study was to evaluate the safety and tolerance of consumption of infant formula supplemented with MFGM, compared to a standard formula (SF), and a breastfed group reference (BFR) in the first year of life.

## Materials and methods

### Ethical approval

The present study was approved by the Medical Research Ethics Committee of Xiamen University (Ethic No. 20150817); and Maternal & Child Health Hospital of Fuzhou; Maternal & Child Health Hospital of Fuqing; 2^nd^ Fuzhou Hospital; and Changle Hospital (Ethics No. 20151111, 20,151,110, 20,151,110, 20,160,307 respectively). This trial was registered at the Australian New Zealand Clinical Trials Registry (ANZCTR) with Trial registration number ANZCTR12616001571460 on 14/11/2016. This study was conducted in accordance with the Declaration of Helsinki and its subsequent amendments. Written informed consent was obtained from the parents or caregivers of all infants before enrolment and randomization.

### Study design

**Fig. 1:**
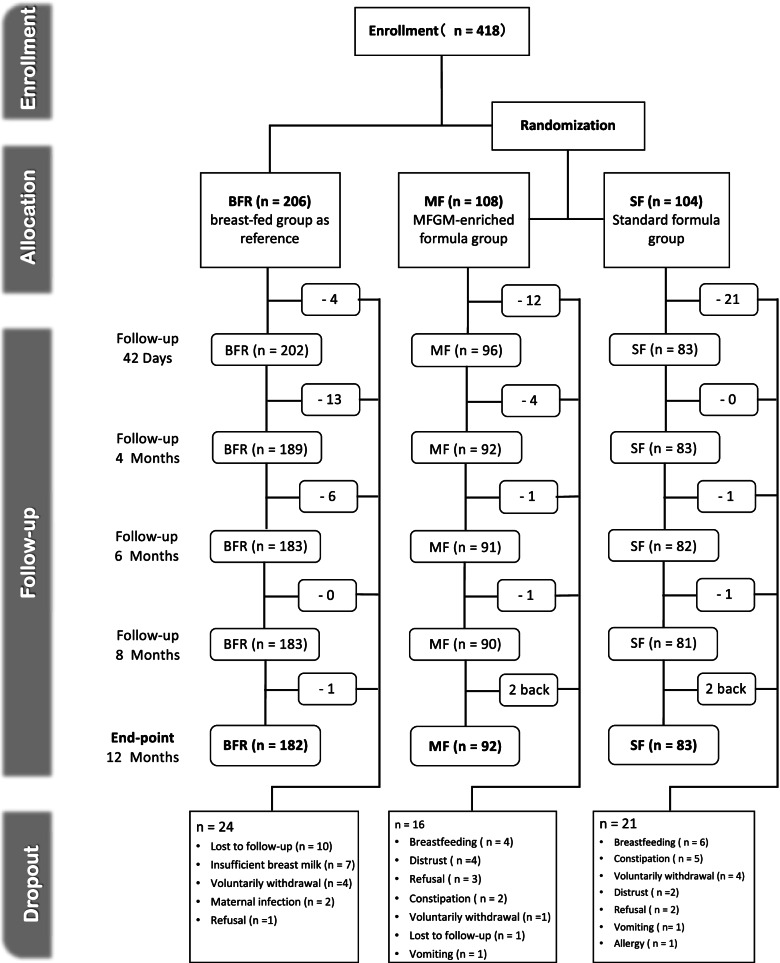
Flow chart of change in infant numbers in the MF, SF, and BFR groups during the study period and dropout reasons. MF, MFGM-enriched formula; SF, standard formula; BFR, breast-fed reference

The present study was a multicenter, randomized, double-blind, parallel group, controlled trial stratified for gender to evaluate the safety and tolerance of MF, carried out in Fuzhou, China, described in detail in our recent report [24]. Parents/caregivers were advised to feed the assigned infant formula to their baby based on age; or to exclusively breastfeed at least to 0- 6 months of age, as well as to record and report any medication or treatment initiated throughout the trial and/or any other concomitant food consumed by the infant at each visit at 42 days ± 5d (V2), 4 months ± 5d (V3), 6 months ± 5 days (V4), 8 months ± 5 days (V5) and 12 months ± 5 days (V6) of age (Fig. 1). Infants were allowed to be introduced to complementary foods from 4 months of age, but they continued to be fed their allocated formulas at least 60% of total food intake from 4 to 6 months of age until the end of the study.

Physical examination, growth measurements, 24 h dietary recall of food intake and tolerance measures, illness symptoms and medication use recorded in a diary were collected at each visit throughout the follow-up schedule by the same pediatrician at each study site. The two formula-fed groups and reference group received standard postnatal care.

### Study population

As described previously [12], 212 formula-fed infants (formula-fed > 60%) were recruited within 14 days postpartum from January 2016 to October 2016 and randomized to receive (MF, *n* = 108) or standard formula (SF, *n* = 104). Sample size was based on a power analysis to find a difference of 0.5 SD in the primary outcome [24]. At the same time, an exclusive breastfeeding group were recruited within 14 days postpartum (*n* = 206). Inclusion criteria were healthy newborn regardless of mode of delivery, gestational age at birth 36 to 42 weeks, birth weight 2500 to 4000 g, and mother having the intention to breastfeed (> 90% up to 6 months) or formula-feed (> 60%) until 6 months.

### Composition of infant formulas

The MF and SF were powdered formulas intended for full nutritional support of infants from birth to 6 months (stage 1) and 6 months—12 months (stage 2). Apart from the levels of MFGM components, the standard and experimental formulas had identical composition (Table 1). The two infant formulas were produced and supplied by the Fonterra Co-operative Group Ltd, New Zealand.

**Table 1:** Demographic and baseline characteristics of the intent-to-treat population

	MFGM-enriched formula (*n* = 108)	Standard-formula (*n* = 104)	P	Breast-fed Reference (*n* = 206)	P
			MF vs SF		Overall
Male, n (%)	53 (49.1)	49 (47.1)	1.00	103 (50.0)	0.89
Caesarean birth (%)	45 (41.7)	36 (34.6)	0.90	67 (32.5)	0.17
Any sibling (%)	74 (68.5)	72 (66.3)	1.00	107 (51.9)	**0.002**
Maternal disease during pregnancy (%)	7 (6.4)	9 (8.6)	1.00	12 (5.8)	0.90
Weight at birth ^1^(g)	3206.9 ± 356.6	3241.9 ± 394.0	1.00	3284.1 ± 334.6	0.18
Length at birth (cm)	49.5 ± 1.7	49.6 ± 1.9	1.00	49.5 ± 1.4	0.85
BMI at birth (kg/m2)	13.1 ± 1.2	13.2 ± 1.3	1.00	13.4 ± 1.1	0.08
Head circumference at birth (cm)	33.9 ± 1.2	33.9 ± 1.4	1.00	34.2 ± 1.2	0.09
Any disease after delivery (%)	7 (6.5)	4 (3.8)	0.68	0 (0.0)	**0.002**
Maternal age (y)	29.4 ± 5.0	29.7 ± 5.0	1.00	27.7 ± 4.6	**0.001**

^1^ Data are means ± standard deviation or number (%). A general linear model, group and sex as fixed effects, group by sex interaction and site as a random factor

### Dietary intake

The participants received formula milk powder according to the coding in the concealed envelopes along with instructions for milk preparation and infant feeding. In the 2^nd^ week of enrolment all parents received a telephone call or home visit from the doctors or research nurses of each site to check milk intake, milk tolerance and to provide some advice on infant feeding including the amount of milk (milliliters) that should be prepared per feed, the number of milk feeds per day, and to record the volume of residual milk (milliliters) left in the bottle after feeding. A 24-h recall of food intake, tolerance, and stool characteristics were collected at each time visit (Fig. 1). To improve study adherence, participants in the formula-fed groups were asked to return the empty formula cans at each clinic visit. The counts of incoming empty formula cans and outgoing formula milk cans were recorded by the clerk in charge of the formula storage house. Parents or caregivers of breastfed infants were required to keep a record of all formula fed to their infants, so that researchers could check compliance with breast milk intake.

Evaluation of digestive tolerance was based on the volume of formula intake and any other dietary intakes, stool characteristics, including frequency of predominant stool color (brown, yellow, green, red or black) and consistency for each stool (1. hard (lumps), 2. formed (normal), 3. soft (creamy), 4. liquid or loose/watery (like diarrhea)), diarrhea defined as 4 or more loose or watery stools in 24 h and mucus, frequency of spitting up or vomiting, crying after 15 min feeding, night crying and sleep behavior and periods of restlessness.

### Assessment of adverse events and health

Medically confirmed adverse events (AEs) including onset, duration, severity and seriousness, relationship with the study formula, any actions taken, and the outcomes were collected during the study. AEs were considered to be serious (SAEs) if they were fatal or life-threatening events causing permanent harm or requiring or extending inpatient treatment at a hospital. Serious AEs were followed-up by the investigator until a stable situation had been reached. The study investigators assessed the seriousness of an AE and its causal relation to the study products. All events that were anticipated from the study population were documented as AEs in the study database.

### Assessment of growth

The anthropometric measures including body weight, length and head circumference were recorded at baseline, 42 ± 5 days and 4, 6, 8, and 12 months ± 5 days of age respectively (Fig. 1). The outcome of the weight gain (g/day) in the enrolled infants up to 4 months of age was calculated based on the difference in infant weight between the baseline visit and the visit at the age of 4 months, divided by the number of days between these two visits to cover the period of exclusively feeding with the test formulas. Weight of infants was measured to the nearest 10 g (HW-B70, Xiamen Zhonghenkang Technology Co. Ltd, China), recumbent length (Lejia HW-B70, Henan China) and head circumference was measured to the nearest 1 mm [12].

### Statistical analysis

All statistical analyses were performed using IBM SPSS V22.0 Statistics (SPSS, Inc., Chicago, IL, USA). Comparisons of the means of baseline characteristics between the 2 randomized groups (MF and SF) were completed by independent samples t test. The comparison of mean frequency of stool color and consistency was analysed using a generalized linear model binary logistic regression for the groups at six time points. And the comparisons between means of milk intake, digestive tolerance including frequency of stool mucus, milk vomiting, crying after feeding, night crying and incidence of AEs between three groups at six individual time points were performed using a general linear model ANOVA with Bonferroni’s adjustment for post hoc paired comparisons. Longitudinal analyses of weight (gain), recumbent length, head circumference and BMI from enrolment to 12 months of age between the groups were performed with the use of general linear model repeated measures analysis of variance (ANOVA) with six time points, adjusted for group and sex as a fixed factor, group by sex interaction, and site as a random factor. To investigate potential different time trends within each group, we included the interaction between time and group in the model. All analyses were conducted on an intention-to-treat basis, where cases were included in the analysis irrespective of compliance with the intervention. Results are expressed as mean ± SEM, and a significance level of 0.05 was used.

The correlation between infant growth outcomes and stool characteristics were analyzed by converted the stool characteristics into numeric, e.g. stool color blackish green = 1, green = 2, light yellow = 3 and golden = 4 for the correlation between two numeric measures (e.g. body weight and stool color) using Pearson correlation analysis. Since this is a multivariable correlation analysis, significance was set at the 0.01 level (2 tails). All statistical analyses were completed with the use of IMB SPSS 22·0 Statistics (SPSS, Inc., Chicago, IL, USA).

## Results

### Subjects

There were no significant differences in body weight, recumbent length, head circumference and BMI at birth between three groups. The mothers in the MF and SF group were 1.7–1.9 years older than those in the BFR group (*P* < 0.001) and the percentages of infants with a sibling in the BFR, MF and SF were 51.9% 68.5% and 66.3% (*P* = 0.002) respectively. All baseline characteristics between the two formula-fed infant groups were not significantly different (Table 2). All infants (*n* = 418) were followed-up from enrolment at postnatal day 0–14 to 12 months of age. Sixteen infants (14.81%) from the MF, 21 infants (20.19%) from the SF, and 24 infants (11.65%) from BFR group were withdrawn or lost to follow-up before the end of the study (Fig. 1).

**Table 2:** Phospholipid and gangliosides content in the MFGM of reconstituted study formulas ^1^

Nutrient per 100 mL	MFGM IF	Control IF	MFGM FOF	Control FOF
MFGM components				
Phospholipids (mg)	71.5	39.4	75.5	36.5
Phosphatidylcholine (mg)	20.6	11.0	21.3	10.3
Phosphatidylethanolamine (mg)	19.1	9.6	20.4	9.1
Phosphatidylinositol (mg)	6.9	4.5	6.9	3.9
Phosphatidylserine (mg)	6.7	3.3	7.2	3.2
Sphingomyelin (mg)	12.8	6.3	13.3	5.9
Gangliosides (mg) ^2^	2.5	1.4	2.7	1.5

^1^ Due to batch-to-batch variation and the necessity of producing 2 batches during the study

^2^Measured as GD3 as described in Fong et al. (2011)

### Formula and complementary food intake

**Fig. 2:**
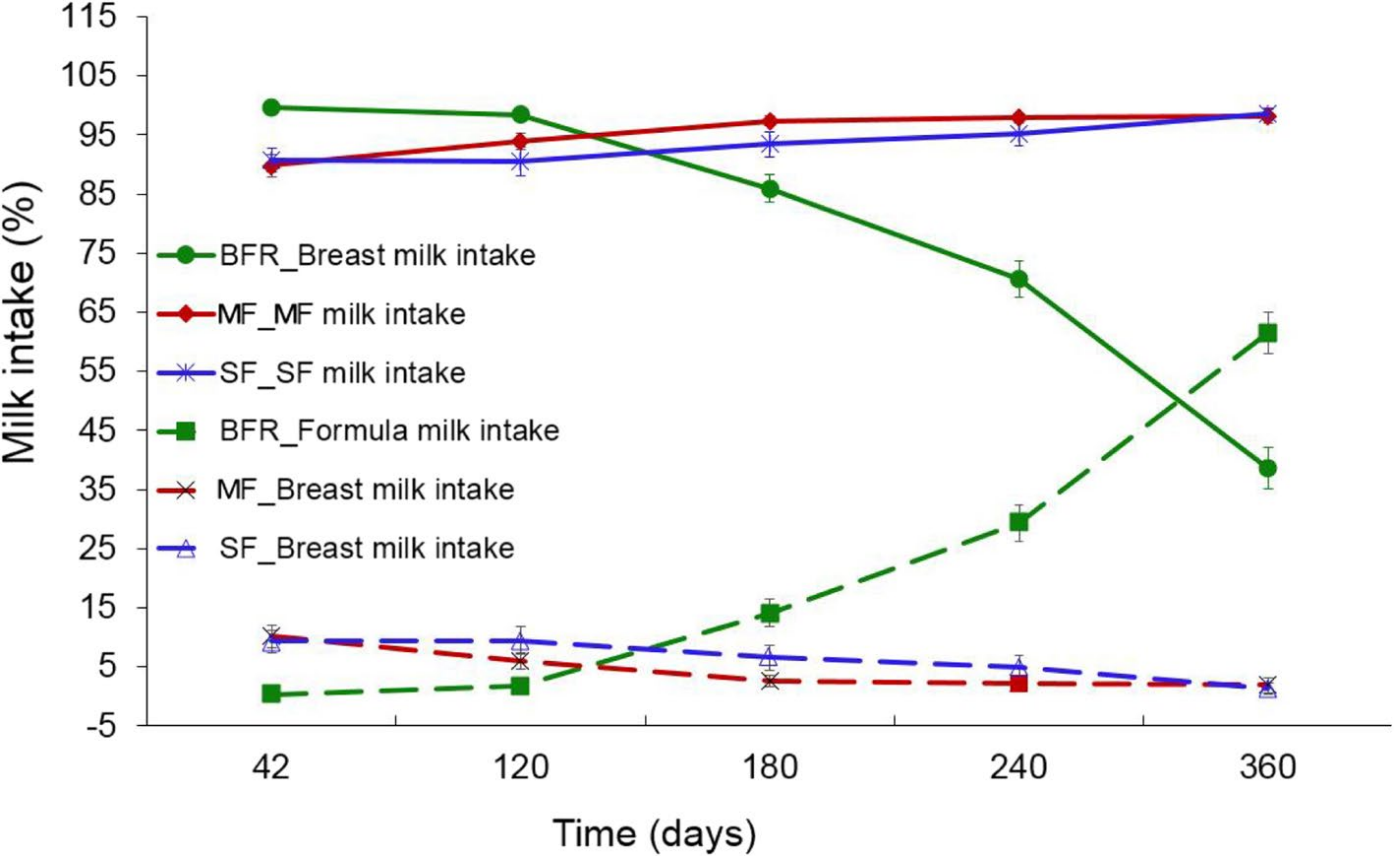
Mean (95% CI) of percentage milk intake (volume) of study formula in the MF (solid red line); and SF (dashed dot blue) and breast milk in BFR group (dashed blue line) at the postnatal age of 42 days, 4 months, 6 months, 8 months, and 12 months. MF: MFGM-enriched formula; SF: standard formula, BFR: breastfed reference

There was no significant difference in formula milk intake between the MF and the SF group throughout study (Fig. 2). The mean volume of formula intake in the two formula-fed groups was above 90% and 93% of assigned formula milk in the first 4 and 6 months of life respectively (Fig. 2) and was increased slightly to ~ 98% during the 7–12 month period compared to the first 6 months of formula volume intake (Fig. 2). In the BFR group, however, most were consuming solely breast milk (~ 100%) as a source of nutrition during the first 4 months of life. From the age of 4 months to 6 months, exclusive breast milk intake was more than 85% in the BFR group. Breast milk intake in the BFR group gradually decreased from 85% at 6 months to 70% and 40% at 8 and 12 months respectively (Fig. 2).

**Fig. 3:**
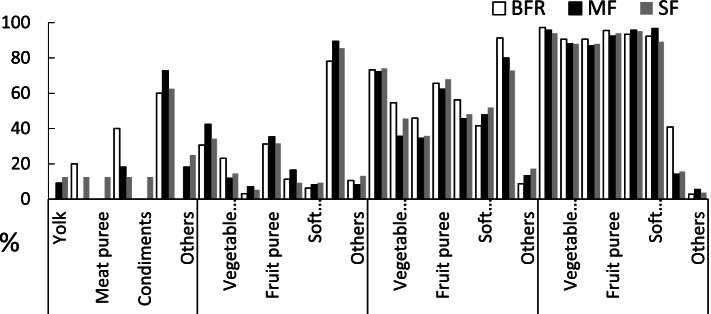
Frequency (%) of different type of complementary food intakes at different age of infants who were introduced complementary food by parents/caregivers at 4 months (*n* = 24), 6 months (*n* = 321), 8 months (*n* = 354), 12 months (*n* = 356) respectively. MF: MFGM-enriched formula (black column); SF: standard formula (grey column), BFR: breastfed reference (white column)

At the 4-month visit, 2.65%, 12.09% and 9.64% of infants in the BFR, MF and SF groups were introduced with complementary foods, such as rice paste or soup, and fruit puree (Table 3). At the 6-month visit however, the percentage of infants in the BFR, MF and SF group introduced with complementary foods increased to 87.43%, 93.41% and 92.68% respectively (Table 3). The major complementary foods at this age included egg yolk, rice paste or soup, fruit and vegetable puree (*P* > 0.05, Fig. 3). All infants received complementary foods from 8 to 12 months, including meat puree, egg yolk, and soft rice and noodle soups. There were no significant differences in the rate of introduction of complementary foods at different ages, nor in the variety of complementary foods between the MF and SF group or among the three groups (*P* > 0.05, Fig. 3).

**Table 3:** Comparison of the percentage of complementary food intake between three groups of infants at different ages

Month	Group	Number	Total	Rate	MF VS SF	Overall *P* value
4 M	BAR	5	189	2.7%		
	MF	11	91	12.1%	*P* = 0.53	*P* = 0.07
	SF	8	83	9.6%		
6 M	BFR	160	183	87.4%		
	MF	85	91	93.4%	*P* = 0.85	*P* = 0.34
	SF	76	82	92.7%		
8 M	BFR	183	183	100%		
	MF	90	90	100%	-	-
	SF	81	81	100%		
12 M	BFR	181	181	100%		
	MF	92	92	100%	-	-
	SF	83	83	100%		

The main effects between groups were analysed using a general linear mixed model with adjusted for group and sex as a fixed effect, group and time interaction and site as a random effect. *MF* MFGM-enriched formula, *SF* standard formula, *BFR* breast-fed reference

### Digestive tolerance

**Fig. 4:**
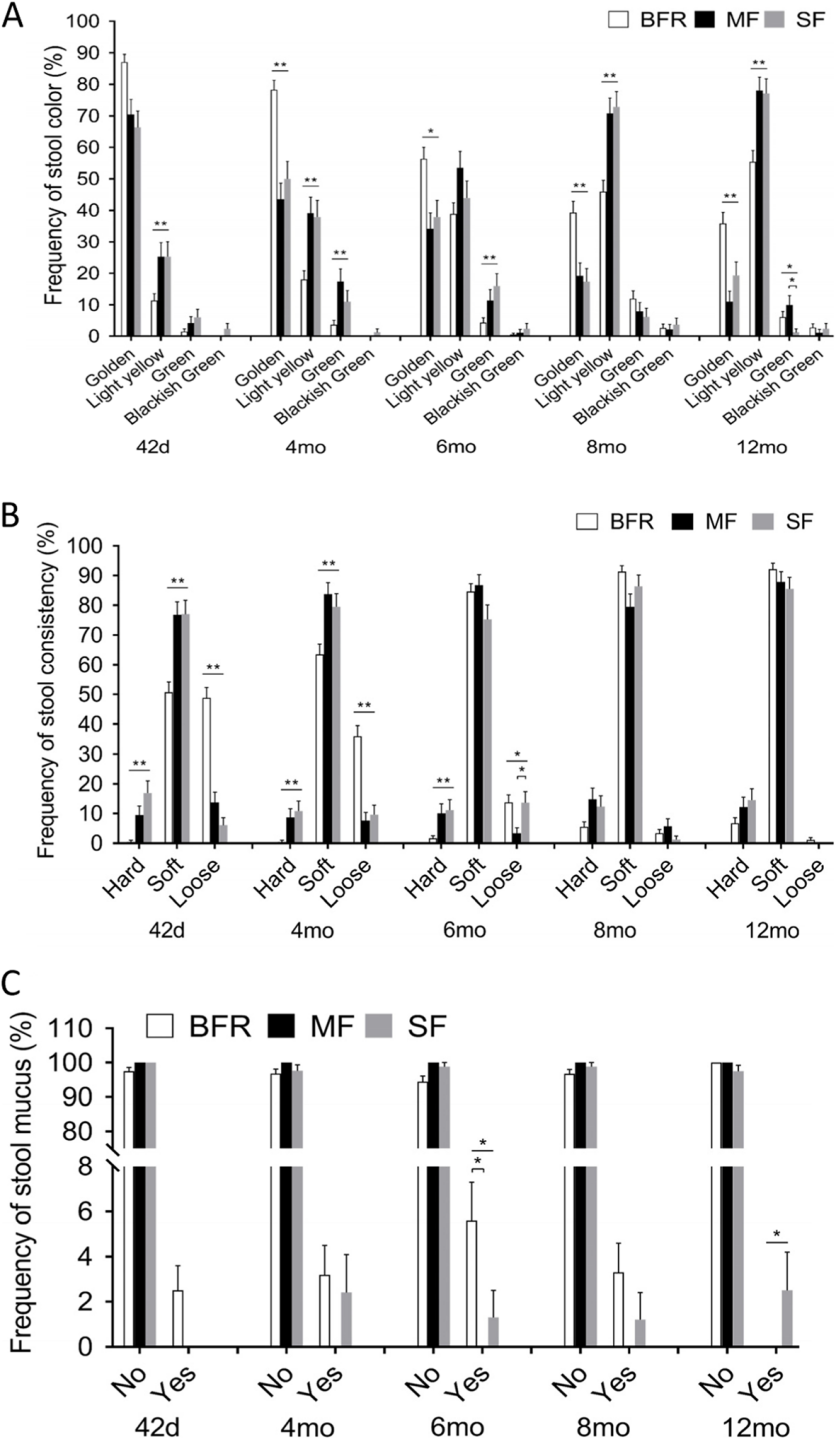
Comparison of incidence of stool colors, consistency and stool mucus presented as mean percentages of total occurrences in the MF group (black column) and SF group (grey column), and BFR group (white column) at the postnatal age of 42 days, 4 months, 6 months, 8 months, and 12 months. A. Frequency of stool colors between the groups. B. Frequency of stool consistency between the groups, C. Frequency of stool mucus between the groups. *p < 0.05, **P < 0.005. MF: MFGM-enriched formula; SF: standard formula; BFR: breast-fed reference

The stool color in all three groups gradually and significantly changed from golden to light yellow during the study (Fig. 4A, *P* < 0.05). There were no significant differences in frequency of stool color changes between two formula-fed groups throughout the study, with the exception that the MF group had significantly higher frequency of green stools at 12 months (odds ratio 8.92, *P* = 0.040, Fig. 4A). However, the two formula-fed groups had significantly lower frequency of golden stool and higher frequency of light-yellow stool compared with the BFR group over the first year of age (odds ratio 0.21–0.47, *P* < 0.05–0.001). The two formula-fed groups also had significantly higher frequency of green stool compared with the BFR group at 4 months and 6 months (odds ratio 2.78–5.47, *P* < 0.05–0.001, Fig. 4A), but these differences were no longer apparent at 8 months (*P* > 0.05). There was a low frequency of blackish green stools across all groups throughout the study. The rate of change of stool color in all three groups was relatively faster from 42 days to 8 months, and slower from 8 and 12 months of age (Fig. 4A).

Soft stool was the predominant stool pattern (50–95%) among the 3 groups of infants throughout the first year of life. There were no significant differences in the frequency of soft stools between the two formula-fed groups throughout the study, However, the frequency of soft stool in both SF and MF was higher than that for the BFR group at 42 days and 4 months of age (Odds ratio 2.95–3.27, *P* < 0.01, Fig. 4B). The BFR group had a significantly higher frequency of loose stools at 42 days and 4 months compared with the two formula-fed groups (Odds ratio 1.01–14.93, *P* < 0.01), but this frequency declined to similar levels in the SF group by 6 months and then reached the same levels as the two formula-fed groups by 8 months of age (*P* > 0.05, Fig. 4B). Furthermore, frequency of loose stools in the MF group was 50% at the first 42 days (*P* > 0.05), and changed to 85% “low frequency” at 6 months (odds ratio 0.22, *P* = 0.023, Fig. 4B) compared to the SF group. The two formula-fed groups, in particular the SF group had significantly higher frequency of hard stools compared to the BFR group from birth to 6 months (Odds ratio 6.6–41.76, *P* < 0.001, Fig. 4B), but not at 8 and 12 months (*P* > 0.05, Fig. 4B).

Stool mucus was not observed in the MF group throughout the study, nor in the SF group at 42 days or the BFR at 12 months (Fig. 4C). The frequency of stool mucus in the SF group from 4–12 months was 1.2%-2.5%. The frequency of stool mucus in BFR group was about 2.1–2.5% between 42 days and 8 months of age (Fig. 4C). The overall difference in frequency of stool mucus was not significant between the 3 groups (*P* > 0.05), except at 6 months of age, when BFR group had significant higher frequency of stool mucus than MF group (95% CI 0.002–0.110, *P* = 0.039, Fig. 4C) and lower than SF group at 12 months of age (95% CI -0.048–0.000, *P* = 0.046, Fig. 4C).

**Fig. 5:**
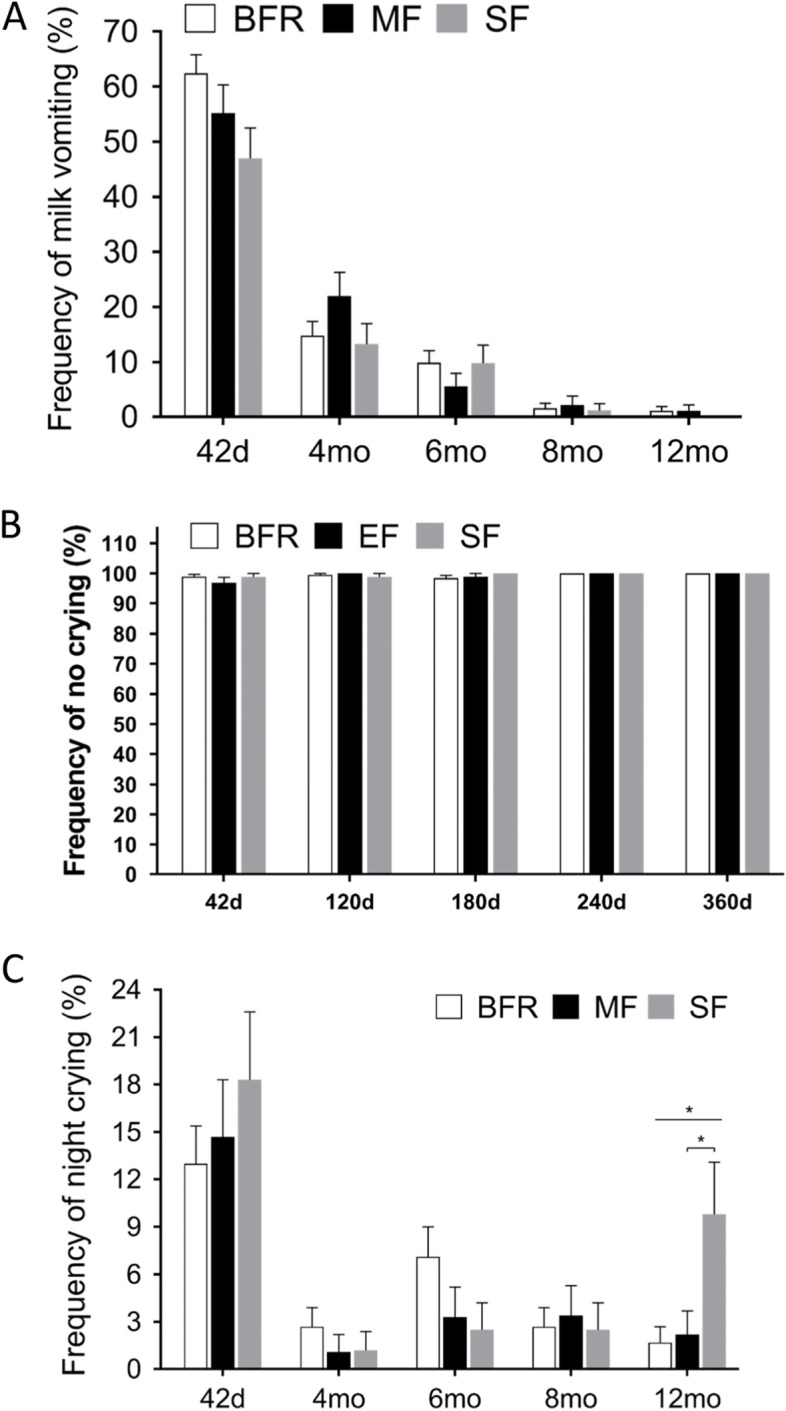
Comparison of frequency of milk vomiting, no crying within 15 min after feeding and night crying presented as mean percentages of total occurrences in the MF group (black column), SF group (grey column) and the BFR group (white column) at the postnatal age of 42 days, 4 months, 6 months, 8 months, and 12 months. A. Frequency of milk vomiting between the group; B. Frequency of no crying within 15 min after feeding, C. Frequency of night crying between the groups. *p < 0.05, MF: MFGM-enriched formula; SF: standard formula; BFR: breast-fed reference

The frequency of milk spit-up/vomiting decreased over the course of the study in all three groups, as reported by parents or infant caregivers through a 24 h feeding recall per visit (Fig. 5A). There was no significant difference in the frequency of milk spit-up/vomiting among the groups at any time point (*P* > 0.05, Fig. 5A), though the SF group had a relatively lower incidence of milk spit-up/vomiting compared with the BFR group at 42 days of age and the MF group at 4 months had a slightly higher incidence of milk spit-up/vomiting compared to the other two groups (*P* > 0.05). The frequency of crying within 15 min after feeding among the three groups was similar throughout the study (Fig. 5B). The frequency of night crying decreased significantly from postnatal age of 42 days to 4 months and remained stable during the rest of the study (Fig. 5C). In 12-month-old infants, however, the SF group had significantly higher frequency of night crying compared to the MF group and the BFR group (95% CI 0.008–0.144 and 0.021–0.141, *P* = 0.023 and 0.003**,** Fig. 5C).

### Adverse events

Prevalence of AEs from 42 days to 12 months between the groups is shown in Table 4.

**Table 4:** Incidence of adverse events at age of 42 days, 4, 6, 8 and 12 months between the MFGM-enriched formula (MF), standard formula (SF) and breast-fed reference (BFR) groups of infants

**Age**	**Adverse events**	MF (*n* = 108)	SF (*n* = 104)	***P* value (MF vs SF)**	BFR (*n* = 206)	**Overall-*P* value**
42-day BFR (*n* = 202); MF (*n* = 96); SF (*n* = 83)	Total	2 (2.08) ^1^	3 (3.61)	0.66	6 (2.97)	0.83
	Skin rashes	3 (3.13)	0 (0.00)	0.25	10 (4.95)	0.11
	A Hordeolum	0 (0.00)	0 (0.00)	-	1 (0.50)	0.64
	Upper respiratory infection ^2^	0 (0.00)	0 (0.00)	-	3 (1.49)	0.26
	Lower respiratory infection ^3^	1 (1.04)	1 (1.20)	1.00	1 (0.50)	0.78
	Constipation	1 (1.04)	2 (2.41)	0.60	0 (0.00)	0.11
	Diarrhoea	0 (0.00)	0 (0.00)	-	1 (0.50)	0.64
	Fever > 38 °C (episodes)	0(0.00)	0 (0.00)	-	1(0.50)	0.64
	Days with fever	/	/	-	7	-
4-month BFR (*n* = 189); MF (*n* = 92); SF (*n* = 83)	Total	8 (8.70)	3 (3.61)	0.22	7 (3.70)	0.16
	Skin rashes	1 (1.09)	0 (0.00)	1.00	1 (0.53)	0.62
	Pyrexia	1 (1.09)	0 (0.00)	1.00	3 (1.59)	0.51
	Upper respiratory infection^2^	3 (3.26)	1 (1.20)	0.62	8 (4.23)	0.44
	Lower respiratory infection^3^	0 (0.00)	1 (1.20)	0.47	0 (0.00)	0.18
	Constipation	2 (2.17)	0 (0.00)	0.50	0 (0.00)	0.051
	Diarrhoea	1 (1.09)	1 (1.20)	1.00	2 (1.06)	0.99
	Fever > 38 °C (episodes)	1 (1.09)	0 (0.00)	1.00	0 (0.00)	0.23
	Days with fever	10	/	-	/	
6-month BFR (*n* = 183); MF (*n* = 91); SF (*n* = 82)	Total	14 (15.38)	6 (7.32)	0.15	11 (6.01)	**0.031**
	Skin rashes	0 (0.00)	0 (0.00)	-	1 (0.55)	0.62
	Pyrexia	4 (4.40)	2 (2.44)	0.69	5 (2.73)	0.70
	Upper respiratory infection^2^	4 (4.40)	0 (0.00)	0.12	4 (2.19)	0.15
	Lower respiratory infection^3^	7 (7.69)	3 (3.66)	0.34	0 (0.00)	**0.001**
	Diarrhoea	1 (1.10)	2 (2.44)	0.60	2 (1.09)	0.66
	Fever > 38 °C (episodes)	2(2.20)	0 (0.00)	0.50	2 (1.09)	0.39
	Days with fever	/	/	-	/	-
8-month BFR (*n* = 183); MF (*n* = 90); SF (*n* = 81)	Total	11 (12.22)	18 (22.22)	0.10	32 (17.49)	0.22
	Gastroenteritis	0 (0.00)	1 (1.23)	0.47	0 (0.00)	0.19
	Skin rashes	1 (1.11)	0 (0.00)	1.00	0 (0.00)	0.23
	Pyrexia	4 (4.44)	4 (4.94)	1.00	14 (7.65)	0.51
	Upper respiratory infection^2^	5 (5.56)	7 (8.64)	0.55	28 (15.30)	**0.040**
	Lower respiratory infection^3^	5 (5.56)	6 (7.41)	0.76	2 (1.09)	**0.023**
	Diarrhea	1 (1.11)	5 (6.17)	0.10	2 (1.09)	**0.026**
	Fever > 38 °C (episodes)	0 (0.00)	0 (0.00)	-	4 (2.19)	0.15
	Days with fever	/	/	-	/	-
12-month BFR (*n* = 182); MF (*n* = 92); SF (*n* = 83)	Total	25 (27.17)	19 (22.89)	0.60	41 (22.53)	0.68
	Hand-foot-and-mouth disease	0 (0.00)	1 (1.20)	0.47	0 (0.00)	0.19
	Gastroenteritis	1 (1.09)	0 (0.00)	1.00	0 (0.00)	0.24
	Skin rashes	0 (0.00)	1 (1.20)	0.47	2 (1.10)	0.59
	Pyrexia	16 (17.39)	13 (15.66)	0.84	24 (13.19)	0.63
	Upper respiratory infection^2^	17 (18.48)	9 (10.84)	0.20	23 (12.64)	0.28
	Lower respiratory infection^3^	3 (3.26)	1 (1.20)	0.62	6 (3.30)	0.60
	Constipation	1 (1.09)	0 (0.00)	1.00	0 (0.00)	0.24
	Diarrhoea	3 (3.26)	3 (3.61)	1.00	5 (2.75)	0.93
	Fever > 38 °C (episodes)	5 (5.43)	1 (1.20)	0.21	10 (5.49)	0.26
	Days with fever	/	/		/	

^1^ Data in the brackets show percentage

^2^ Upper respiratory infection: an infection that affects the nasal passages and throat

^3^ Lower respiratory infection: These include bronchitis and pneumonia with using antibiotics

During the study, 8 types of AEs were reported from 212 formula-fed infants (skin rashes, upper respiratory infection, lower respiratory infection, constipation, diarrhea, pyrexia, gastroenteritis and hand-foot-and-mouth disease). Of these, 7 types of AEs in the MF, all 8 types in the SF groups, and 6 types in the BFR group were reported by the parents/caregivers (Table 4). Adverse event and morbidity rates were similar between MF and SF throughout study. Constipation was the most common reason for early dropout in two formula-fed groups. Over the 0–6-month period, 2 infants from the MF and 5 infants from SF group dropped out for this reason, but it was not reported for any of the BFR infants. At the 6-month assessment, however, the MF group had more AEs (15.38%) compared with the BFR group (6.01%) and SF (7.32%) (95% CI 0.013–0.049, *P* = 0.031, Table 4). A significantly lower incidence of lower respiratory infections (0% and 1.09%) was reported in the BFR group compared to the MF (7.69% and 5.56%) and SF (3.66% and 7.41%) at the 6 (95% CI 0.000–0.008, *P* = 0.001) and 8 (95% CI 0.000–0018, *P* = 0.023) month visits, respectively (Table 4). One unexpected finding was that the rate of upper respiratory infection in BFR was higher than for the MF and SF groups at the 8-month visit (95% CI 0.007–0.038, *P* = 0.040). At this time point, the SF group had significantly higher prevalence of diarrheal events (6.17%) compared with the MF (1.11%) and BFR (1.09%) (95% CI 0.011–0.046, *P* = 0.026, Table 4). At the 12-month visit, there were no differences in AEs among three groups (*P* > 0.05) though pyrexia and diarrhoea were more common in both formula groups than in the BFR group (Table 4). Only one infant, in the BFR group, suffered an SAE (febrile convulsion that required hospitalization).

### Growth

All growth parameters for the 3 infant groups were within the normal range. All infants were exclusively fed formulas or breast milk from birth to 4 months of age before introducing complementary foods. Therefore, we analyzed infant growth characteristics during this period. The mean (± SD) weight gain and changes in recumbent length, head circumference, and body mass index (BMI) over this 4-month period is summarized in Table 5. There were no significant differences in mean growth rate between the MF (32.99 ± 6.0 g/d), SF (32.38 ± 5.99 g/d), and BFR (31.36 ± 5.63 g/d) groups over the 0–4 month period (*P* = 0.09, Table 5). This was not altered when male and female data were analyzed separately. Mean changes in recumbent length and head circumference measurements from 0–4 months were not significantly different between the two formula-fed groups (*P* > 0.05, Table 5), nor when all three groups were included in the analysis (*P* > 0.05 Table 5).

**Table 5:** Weight gain and increases in recumbent length, head circumference, and body mass index (BMI) between three groups of infants during **0 – 4 months** of age

		MFGM-enriched formula			Standard formula			P ^1^	Breast-fed reference			P ^1^
	Gender	n	Mean	SD	n	Mean	SD	MF vs SF	n	Mean	SD	Overall
Weight (g/d)		206	32.99	6.07	108	32.38	5.99	0.63	104	31.36	5.63	**0.09**
	M ^1^	103	35.15	6.06	53	35.76	4.14	0.23	49	33.63	5.59	0.19
	F ^2^	103	30.79	5.24	55	29.13	5.68	1.00	55	29.15	4.72	0.11
Recumbent length (mm/month)		206	36.54	4.96	108	35.94	5.36	1.00	104	35.87	5.16	0.51
	M ^1^	103	38.42	4.35	53	38.12	4.79	1.00	49	38.05	4.44	0.88
	F ^2^	103	34.60	4.79	55	33.86	5.04	1.00	55	33.74	4.92	0.55
Head circumference (mm/month)		206	16.99	3.47	108	18.26	9.72	1.00	104	17.66	3.98	0.18
	M ^1^	103	17.80	3.34	53	20.79	3.39	0.59	49	18.77	3.88	0.07
	F ^2^	103	16.16	3.41	55	15.85	3.43	0.98	55	16.58	3.77	0.62
BMI (kg/m^2^/month)		206	1.06	0.41	108	1.06	0.39	0.36	104	0.97	0.38	0.22
	M ^1^	103	1.10	0.42	53	1.18	0.33	0.13	49	1.00	0.43	0.12
	F ^2^	103	1.01	0.39	55	0.96	0.42	1.00	55	0.94	0.32	0.59

^1^*M* males

^2^*F* females

A general linear model, group and sex as fixed effects, group by sex interaction and site as a random factor with post hoc Bonferroni adjusted comparisons

However, the growth rate of BFR (41.98 ± 10.14 g/d) was the highest followed by the MF (38.49 ± 8.85 g/d) and then SF (37.10 ± 8.59 g/d) for the first 0–42 days of age (*P* = 0.002–0.001, Table 6). Again the outcomes were not changed when male (*P* = 0.001) and female (P = 0.022) were analyzed separately. The BMI in BFR was significantly higher than the two formula-fed groups (*P* = 0.008, Table 6) for the first 0–42 days of age, with the differences being the same for males (*P* = 0.026), but not for females (*P* > 0.05). The two formula-fed groups were not significantly different in any growth parameters in the first 42 days growth (*P* > 0.05, Table 6). Thus, the infants in the two formula-fed groups grew faster after 42 days of age, as their growth had caught up with the BFR infants by 4 months of age (Table 5).

**Table 6:** Weight gain and increases in length, head circumference, and body mass index (BMI) between three groups of infants during **0 – 42 days** of age

		MFGM-enriched formula			Standard formula			P^2^	Breast-fed reference			P^2^
	Gender	n	Mean	SD	n	Mean	SD	MF vs SF	n	Mean	SD	Overall
Weight (g/d)		108	38.49	8.85	104	37.10	8.59	0.91	206	41.98	10.14	** < 0.001**
	M	53	42.43	7.80	49	40.16	7.66	0.68	103	46.21	9.55	**0.001**
	F	55	34.56	8.04	55	34.12	8.40	1.00	103	37.83	8.92	**0.022**
Recumbent length (mm/month)		108	43.90	12.70	104	44.17	11.93	1.00	206	46.02	12.19	0.24
	M	53	47.76	12.11	49	47.76	11.96	1.00	103	50.11	12.06	0.42
	F	55	40.03	12.09	55	40.66	10.82	1.00	103	42.01	10.91	0.57
Head circumference (mm/month)		108	23.75	9.86	104	24.63	9.87	1.00	206	24.28	9.91	0.57
	M	53	26.05	10.18	49	25.67	9.91	0.63	103	25.50	10.17	0.27
	F	55	21.44	8.96	55	23.62	9.72	0.84	103	23.08	9.49	0.50
BMI (kg/m^2^/month)		108	1.76	0.92	104	1.58	0.89	0.64	206	1.96	1.01	**0.008**
	M	53	1.89	0.81	49	1.64	1.03	0.69	103	2.13	1.05	**0.026**
	F	55	1.64	1.01	55	1.53	0.73	1.00	103	1.79	0.94	0.26

^1^ A general linear model, group and sex as fixed effects and site as a random factor with post hoc Bonferroni adjusted comparisons when overall comparison was significant

^2^ M males

^3^ *F* females

**Fig. 6:**
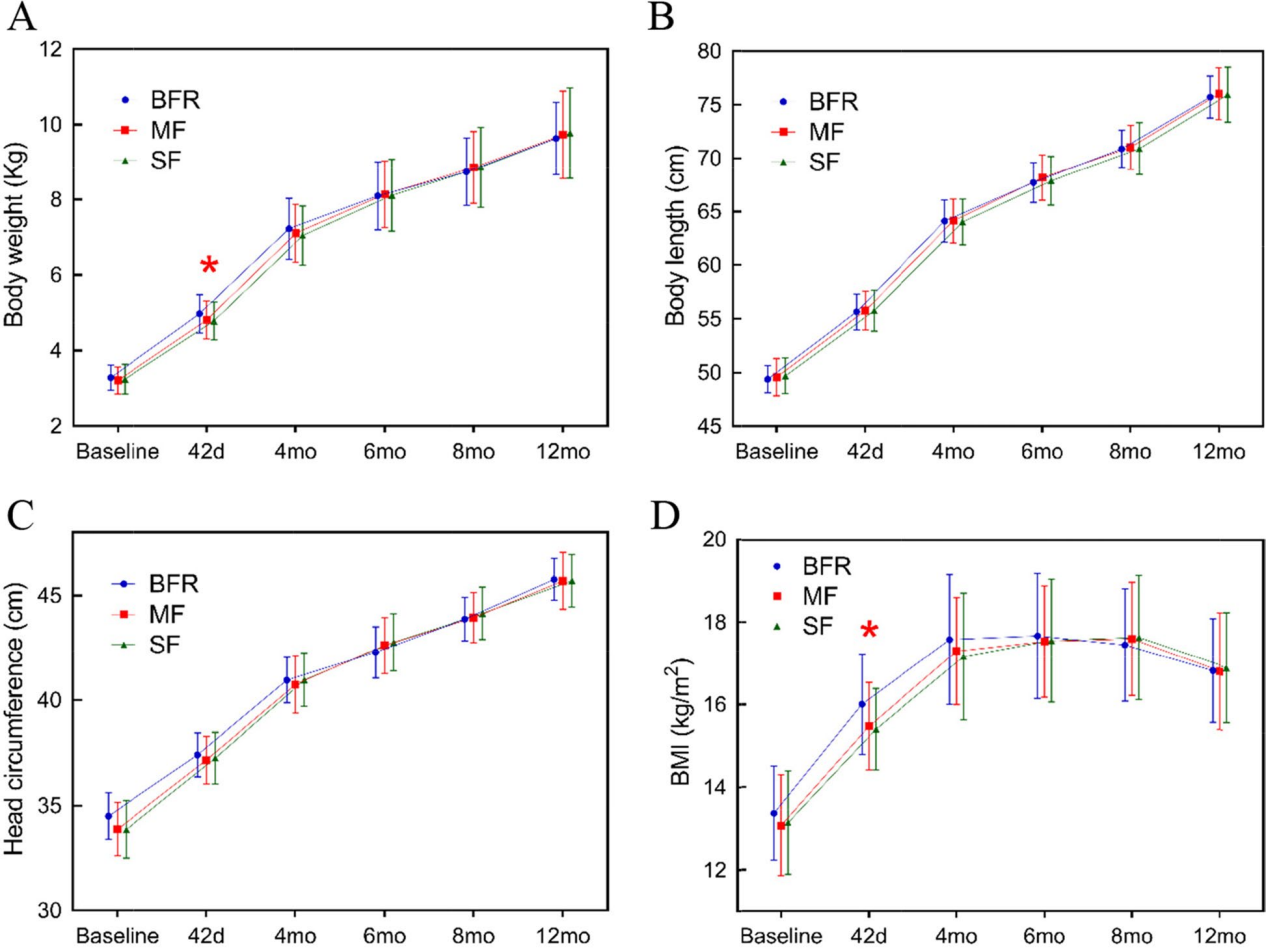
Comparison of mean (± SD) of body weight (A), recumbent length (B), head circumference (C) and body mass index (D) from 0 to 12 months of age in MF (red line, *n* = 108), SF (green line, *n* = 104), and BFR (blue line, *n* = 206) groups. *p < 0.05. MF: MFGM-enriched formula; SF: standard formula; BFR: breast-fed reference; and BMI, body mass index

From 4 to 12 months, the increase in body weight, recumbent length, head circumference and BMI of infants between the 2 formula-fed groups or among the 3 groups were not significantly different (*P* > 0.05, Fig. 6). Overall, there were no significant differences in body weight gain, length, head circumference and BMI of infants between the 2 formula-fed groups nor with the 3-group comparison, for all infants from 4 to 12 months (*P* > 0.05, Fig. 6A-C). Again, these outcomes were not different when male and female cohorts were analysed separately.

### Correlation between infant growth outcomes and stool characteristics

In order to understand whether the infant’s stool characteristics are associated with poor growth outcomes, we analyzed the relationship between variables of stool color, stool consistency and stool mucus with each growth outcome using Pearson correlation. We found there was not significant correlation between infant growth and stool characteristics in BFR (Table 7) and MF group (Table 8). In the SF group however, BMI and frequency of stool consistency was significantly positive corrections (Pearson’s r = 0.368 and 0.346, *P* = 0.003 and 0.004, respectively (Table 9).

**Table 7:** Correlations between infant growth and stool characteristics in breast-fed reference group (BFR) ^1^

		Stool color ^2^		Stool consistency ^3^		Stool mucus ^4^	
		Coefficient	P value	Coefficient	P value	Coefficient	P value
Body weight	42d	-0.065	0.360	0.013	0.855	0.088	0.219
	4mo	-0.111	0.145	0.050	0.515	0.042	0.588
	6mo	-0.068	0.389	-0.118	0.137	-0.062	0.443
	8mo	0.033	0.675	-0.047	0.552	-0.094	0.237
	12mo	0.073	0.363	0.054	0.503	-	-
Body length	42d	-0.132	0.063	0.001	0.990	0.141	0.049
	4mo	-0.093	0.223	0.127	0.095	0.054	0.483
	6mo	-0.080	0.314	-0.034	0.672	-0.063	0.430
	8mo	-0.068	0.394	0.068	0.391	0.031	0.696
	12mo	-0.043	0.592	-0.022	0.782	-	-
Head circumference	42d	-0.037	0.609	0.024	0.740	0.131	0.067
	4mo	-0.083	0.278	-0.020	0.794	0.068	0.374
	6mo	-0.053	0.508	-0.088	0.267	-0.067	0.407
	8mo	-0.065	0.411	-0.118	0.137	-0.072	0.363
	12mo	-0.021	0.797	0.065	0.420	-	-
BMI	42d	0.015	0.837	0.020	0.782	0.014	0.843
	4mo	-0.070	0.361	-0.037	0.627	0.009	0.903
	6mo	-0.030	0.701	-0.122	0.123	-0.029	0.720
	8mo	0.094	0.238	-0.115	0.147	-0.159	0.043
	12mo	0.129	0.107	0.088	0.269	-	-

^1^ Data are processed through Pearson analysis. Correlation is significant at the 0.01 level (2-tailed)

^2^ Stool color: a) blackish green, b) green, c) light yellow and d) golden

^3^ Stool consistency: a) soft, b) hard and c) loose

^4^ Stool mucus: a) no and b) yes

**Table 8:** Correlations between infant growth and stool characteristics in MFGM-formula group (MF)^1^

		Stool color ^2^		Stool consistency ^3^		Stool mucus ^4^	
		Coefficient	*P* value	Coefficient	*P* value	Coefficient	*P* value
Body weight	42d	-0.028	0.802	0.001	0.991	-	-
	4mo	0.075	0.510	-0.031	0.786	-	-
	6mo	-0.122	0.293	0.058	0.612	-	-
	8mo	-0.094	0.420	0.098	0.404	-	-
	12mo	-0.031	0.785	0.113	0.320	-	-
Body length	42d	-0.020	0.858	-0.020	0.859	-	-
	4mo	0.281	0.012	0.110	0.329	-	-
	6mo	0.044	0.706	-0.128	0.265	-	-
	8mo	-0.007	0.955	0.130	0.267	-	-
	12mo	0.064	0.574	0.228	0.042	-	-
Head circumference	42d	0.005	0.966	-0.005	0.962	-	-
	4mo	0.149	0.187	0.061	0.589	-	-
	6mo	0.016	0.888	0.064	0.581	-	-
	8mo	-0.077	0.509	0.163	0.163	-	-
	12mo	-0.065	0.568	0.080	0.479	-	-
BMI	42d	-0.028	0.797	0.022	0.842	-	-
	4mo	-0.134	0.235	-0.140	0.214	-	-
	6mo	-0.211	0.068	0.177	0.121	-	-
	8mo	-0.132	0.255	0.036	0.756	-	-
	12mo	-0.092	0.416	0.009	0.934	-	-

^1^Data are processed through Pearson analysis. Correlation is significant at the 0.01 level (2-tailed)

^2^Stool color: a) blackish green, b) green, c) light yellow and d) golden

^3^Stool consistency: a) soft, b) hard and c) loose

^4^Stool mucus: stool mucus was not found in all infants throughout the trial

**Table 9:** Correlations between infant growth and stool characteristics in standard formula group (SF) ^1^

		Stool color ^2^		Stool consistency ^3^		Stool mucus ^4^	
		Coefficient	*P* value	Coefficient	*P* value	Coefficient	*P* value
Body weight	42d	0.016	0.90	0.156	0.21	-	-
	4mo	-0.047	0.71	0.089	0.47	-0.096	0.44
	6mo	0.023	0.85	0.242	0.06	-0.122	0.34
	8mo	0.093	0.47	-0.025	0.85	-	-
	12mo	-0.215	0.08	0.181	0.14	0.018	0.89
Body length	42d	0.022	0.86	0.177	0.15	-	-
	4mo	-0.022	0.86	0.007	0.95	0.058	0.64
	6mo	0.075	0.55	-0.040	0.76	-0.242	0.05
	8mo	-0.060	0.64	-0.067	0.60	-	-
	12mo	-0.233	0.06	-0.057	0.65	0.092	0.46
Head circumference	42d	0.036	0.77	0.065	0.60	-	-
	4mo	0.008	0.95	0.043	0.73	-0.042	0.74
	6mo	-0.031	0.81	0.115	0.37	-0.154	0.23
	8mo	-0.056	0.66	-0.137	0.28	-	-
	12mo	-0.175	0.16	0.057	0.65	0.012	0.92
Body mass index	42d	0.001	0.99	0.033	0.79	-	-
	4mo	-0.044	0.72	0.101	0.41	-0.156	0.21
	6mo	-0.031	0.80	0.368	**0.003**	0.032	0.80
	8mo	0.185	0.14	0.036	0.780	-	-
	12mo	-0.122	0.33	0.346	**0.004**	-0.045	0.72

^1^ Data are processed through Pearson analysis. Correlation is significant at the 0.01 level (2-tailed)

^2^ Stool color: a) blackish green, b) green, c) light yellow and d) golden

^3^ Stool consistency: a) soft, b) hard and c) loose

^4^ Stool mucus: a) no and b) yes

## Discussion

Human milk is the gold standard for infant nutrition. The MFGM is abundant in human milk, but lacks in standard infant formula, and play an important role in neurodevelopment, cognitive function, intestinal development, maintenance of gut barrier integrity and immune defence [2, 12]. This is the first study on the safety and tolerance of infant formula supplemented only with MFGM in a large sample size in China with a one-year follow-up. Our study of digestive tolerance illustrated through stool colour showed that the BFR group had a higher frequency of golden stool than the formula groups, which is a sign of healthy status of breastfeeding. The MF group had a higher frequency of golden yellow stool at 42 days and at the 8-month visit compared to the SF group (*P* > 0.05). Notably, the acidic environment of gut content will accelerate the infant's intestinal peristalsis, and part of biliverdin is not metabolized into bilirubin by the reduction of intestinal microorganisms, leading to green colored stools. At the 12-month visit, the MF group had a greater frequency of green stools than the SF group (95% CI, odds ratio 8.92 *P* < 0.05). Some possible reasons were that the type of complementary foods, including introduction of green vegetables could impact stool colour or the baby had recently taken additional probiotic supplements (such as bifidobacteria) with strong acid production ability [25] though both study formulas contained a bifidobacteria probiotic [24]. The frequency of soft stools in the MF group was 2.4–11.4% higher than observed in the SF group at 4, 6 and 12 months respectively, indicating that the MF group might have experienced better gut comfort during early growth and development. The difference between the two formula groups was significant at the 6-month visit, when the SF group had a higher frequency of hard or loose stools than the MF group (Fig. 4B), which implies that the digestion adaptability of the SF group was lower than that for the BFR group and the MF group after the provision of complementary food. Thus, MFGM and its components may play a positive role in the early development of the neonatal intestinal tract [26]. The BFR group had a higher frequency of stool mucus in the first 8 months (Fig. 4C), which may be related to the higher content of water and oligosaccharides in breast milk than the two formulas, that is, higher content of water and dietary fiber (oligosaccharides) in breast milk, causing the baby's stool to pass through the intestines relatively quickly.

We also analysed if the stool green and blackish green color were associated with poor growth outcome for the infants using Pearson correlation. The results showed there was a significant relationship between BMI in the SF group at 6 months and 12 months and frequency of stool consistency (*P* < 0.01, Table 9). The results suggest that soft stool has a beneficial effect on growth and the prevalence of loose stools may be associated with a poor growth outcome. Interestingly, the prevalence of loose stools in the BFR was significantly higher than two formula-fed groups of infants (Fig. 4B), which is similar to the results reported by Breij et al. [27]. It might be the parents, and possibly also pediatricians, are more concerned by loose stools in infants fed formula milk compared with breast-fed infants [28]. The intestines naturally secrete mucus to help stools pass more effectively through the intestines. Medical conditions including infections and food allergies can cause mucus in stools, which indicates a degree of dysfunction in the infant's intestinal tract. There was no significant difference in stool mucus between two formula-fed groups. Interestingly, the infants in the BFR group had higher frequency of stool mucus during early development, but it was completely eliminated by the time of the 12-month visit. There was no correlation between stool mucus and poor growth (*P* > 0.01, Table 7). Thus, the results imply that breast milk contains many bioactive compounds that support digestive function such as mucus production to facilitate stools passing in the intestines and to save energy lost during stool elimination process. Thus, in our study population, the mucus is part of a normal process. The characterization of stool mucus in breast-fed infants needs further study. There was no significant correlation between infant growth outcomes and stool characteristics in the MF (*P* > 0.01, Table 8). Overall, our results showed that MFGM enriched formula is closer to the BFR group in the stool characters, especially at 12 months of age (Fig. 4A-C).

The analysis of infant milk spit up/vomiting showed that although the prevalence of milk spit up/vomiting in the BFR group was higher than the two formula-fed group at 42 days, the difference was not statistically significant (*P* > 0.05) [29]. The possible reason is total volume of breast milk ingested during each feeding may be greater for breastfed infants, thus the higher volume may be responsible for the higher frequency of spit-up/vomiting in the BFR group during first months of life. Although infant regurgitation has been reported as the most common functional gastrointestinal disorders across the world [29], this phenomenon had dissipated after 4 months of age in all 3 groups. Similar observations were made in recent publication of Chinese infants [29]. Infant milk spit up/vomiting may be caused by the infant drinking milk too quickly or over-consuming within each feed, as the ring of muscle between the esophagus and the stomach (the lower esophageal sphincter) is not yet fully mature in infants. As long as the infant is healthy, content and growing well, the temporary reflux does not need any medication. Interestingly, there was a trend towards an increase in the frequency of night crying in infants at 6 months compared with 4 months of age, around the age when more complementary food is generally introduced to the infants. It is well known that infant night crying can be caused by discomfort or irritation from a wet or dirty diaper, excessive gas, or pain, e.g. colic. It is perfectly normal for an infant to cry at night when hungry, e.g. infants' habit of preferring night milk intake can also cause them to cry more frequently at night [30] (data not shown). At 12 months of age, the SF group showed a significantly higher frequency of night crying than MF group (*P* < 0.05). Therefore, the night crying might be related to the night milk feeding habit [30] and was associated with a higher frequency of stool mucus at 12 months in the SF group compared to BFR and MF group, as any organic cause of night crying was ruled out by pedistric evaluation. There was no statistical difference in the frequency of all AEs among the three groups on the 42-day, 4-month and 12-month visits (*P* > 0.05). However, the incidence of diarrhea events in the MF and BFR group was significantly lower than the SF group at the 8-month visit (Table 4). Timby et al. [31] described the beneficial effect of MFGM supplementation on diarrhea prevention in infants aged 6–11 months. This period of time is basically consistent with our findings. Our study also demonstrated that the BFR infants had a significantly lower incidence of lower respiratory infections than the two formula-fed group infants at the 6 and 8-month visits (Table 4**)**. Our findings confirm that breastfed infants have fewer infections [32]. Severe AEs occurred in only one case, in the BFR group, during the CLING study, and was thought to be unrelated to formula consumption or breastfeeding.

Our study showed that there was no significant difference in growth rate of body weight, recumbent length, head circumference and BMI between MF group and SF group, or between the BFR group and the two formula-fed groups at 0–4 months or 0–12 months of age. Interestingly, weight gain (g/day) in the BFR group at 42 days of age was significantly higher than that for the two formula-fed groups including both male and female infants (*P* < 0.05, Table 6); the trend of weight gain in the three groups was consistent after 4 months. Thus, our study confirmed that breast milk is the ideal nutrition optimising early neonatal growth and development. Further, no significant differences were observed for the outcome of anthropometric measurement between the two formula-fed groups throughout all follow-up visits, adding additional weight to the hypothesis that the two formulations have a similar efficacy to promote growth.

At 42 days of age, the overall BMI in the BFR group was the highest (1.96), followed by the MF group (1.76) and then the SF group (1.58): the difference between the groups was statistically significant (*P* = 0.008, Table 6). The difference was observed for male (*P* = 0.026), but not for female subjects (*P* = 0.26, Table 6). This result infers that the BMI of the MF group was closer to that of the BFR group, suggesting that MFGM supplementation may help close the gap in early growth rates between breastfed and formula fed infants. The BMI in the three groups showed a slight trend of decline after 6 months of age. The comparison with the standard table of infant’s BMI values showed that the variation trend of BMI in the infants of all 3 groups was in the normal range [33–35]. In addition, the male infants showed a relatively higher growth outcome compared with the female infants for body weight, head circumference and BMI, but overall difference was not significant from 0 to 12 months of age (repeat measure ANOVA Fig. 6). Our findings were similar to previous reports for MFGM-enriched infant formula [31, 36], suggesting that either MF or SF is sufficient to support the normal growth of infants.

## Conclusion

The BFR group had significantly lower AEs of lower respiratory infection (including severe infections) compared with the two formula-fed groups. The growth rates of body weight, recumbent length, head circumference, and BMI of two formula-fed groups were similar to that of the BFR group among Chinese infants during the first year of life, but the growth performance of the BFR group was relatively faster than that for the two formula-fed groups during the first 42 days of postnatal life. The MFGM-enriched formula had a similar digestive tolerance to the standard formula and was different from the breastfeeding in stool colour and consistency score in Chinese infants at 0–6 months of age, a finding that to our knowledge has not been reported previously.

## Data Availability

The datasets that support the conclusions of this article are available by request to the corresponding author. We do not make participants’ data publicly available due to data protection restrictions and participant confidentiality.

## Abbreviations

CLING: Complex Lipids in Neurodevelopment & Growth
MFGM: Milk Fat Globule Membrane
BFR: Breast Fed Reference
MF: MFGM-enriched Formula
SF: Standard Formula
ANZCTR: Australian New Zealand Clinical Trials Registry
